# Medical Opinion and Movement

**Published:** 1911-08-05

**Authors:** 


### Medical Opinion and Movement.

### General Anæsthesia with Reduced Circulation.

At a meeting of the Académie de Médecine, Dr. Delagénière gave an interesting account of his experience of administering a general anæsthetic with the circulation reduced by excluding the blood in the four limbs from the general circulation according to the method devised by Klapp. He has employed this method in 1,179 cases. According to his observations, the anæsthesia obtained in this way is more rapid and requires a much smaller dose of anæsthetic than under ordinary circumstances. The recovery is also more rapid and there is less tendency to vomiting, albuminuria, etc. The chief purpose of the method, however, is to enable one to combat syncope by at once taking off one or more of the constricting bandages around the limbs. The blood previously excluded from the circulation returns and, as it is free from chloroform, it acts as a diluent of the overcharged blood. Moreover, as it is charged with carbonic acid it stimulates the respiratory centre. The chief objection to the method is the liability to phlebitis from constriction of the limbs. The author has observed four cases. The Klapp method, it may be interesting to note, has been tried by several London anæsthetists since it was first reported on.

### Pulmonary Tuberculosis.

Drs. Pedrazzini and Vecchi describe in *Tommasi* a special method of mechano-therapy devised by them for the treatment of pulmonary tuberculosis. Dr. Vecchi was called to treat a case of fracture of the humerus in a man suffering from pulmonary tuberculosis, and observed that in the course of treatment by means of an apparatus to exert traction and reduce the fracture the condition of the lung showed a marked improvement. In consequence of this observation, the author applied a double traction apparatus to a patient with double pulmonary phthisis, and at the same time, in order to help the flow of blood to the lungs, he kept the patient in a recumbent position with the feet higher than the head. At the end of a fortnight the fever completely subsided and the temperature was normal. Since then he has applied the treatment to a dozen cases in conjunction with Dr. Pedrazzini at the Milan Hospital. They only report on seven cases, as the remaining five have not been treated sufficiently long. In these seven cases there was an abundant discharge of bronchial secretion for the first few days, followed by a diminution in expectoration, a fall in temperature, disappearance of sweats, and improvement in the pulmonary signs. The principle of the method is that by placing the body with the feet higher than the head, the viscera pressing on the diaphragm limit the excursions of this organ and the expansion of the bases of the lungs, and, by compensation, cause an increase of ventilation in the apices of the lungs. At the same time an increased flow of blood is brought to the upper part of the chest. Further, this position allows a sort of natural drainage of the bronchial tubes and so facilitates the expectoration of accumulated bronchial secretions and toxines. The traction on the arms enables the patient to use the accessory respiratory muscles without effort and establishes a superior costal respiration instead of a diaphragmatic.

### Hiccough.

Long is the list of remedies that have been recommended for the suppression of obstinate attacks of hiccough, any and every one of which may still fail to relieve in any particular attack. Here, then, are some further means that have been found efficacious in certain cases and are therefore worth recording. Dr. Kanngiesser, of Braunfels, confronted with an attack of hiccough in an old man which failed to yield to all manner of medication, conceived the idea of exerting pressure on the diaphragm by distending the stomach with carbonic acid gas. For this purpose he administered five grammes of citric acid and immediately afterwards an equal quantity of bicarbonate of soda. The hiccough stopped immediately and the patient had several hours' sleep. The following day, however, the hiccough returned, but the same remedy was without effect. Dr. Jödicke, of Stettin, on the other hand, has tried with some success mechanical pressure on the diaphragm by another method. He makes the patient draw up both the legs so as to be fully flexed at the knees and hips, and then holds them pressed hard against the abdominal wall so as to push the viscera as much as possible against the abdomen. He has tried this remedy in several cases and has always obtained immediate relief. Dr. Réthy adopts the idea first suggested by Nothnagel of applying prolonged compression along the whole length of the vertebral column.

### Vaccination by Intestinal Injections.

Drs. Courmont and Rochaix have already demonstrated that guinea-pigs and rabbits can be rendered immune to intravenous inoculations of virulent typhoid bacilli by several injections into the large intestine, by means of a long tube, of 100 c.c. of cultures killed at 53° C. These researches are somewhat difficult to pursue owing to not having virulent typhoid bacilli always at one's disposition. The authors have, therefore, carried out a further series of experiments on the same lines with the bacillus pyocyaneus. They have obtained similar corroborative results with this kind of infection, and they read a paper on the subject at a meeting of the Académie des Sciences. A culture of the bacillus pyocyaneus, aged eight days, was killed by exposure to 60° C. for an hour. Three injections into the lower bowel of rabbits were made at intervals of five days. Each injection consisted of 100 c.c. of the complete culture, containing extra and intra-protoplasmic toxines. The injections were well borne and the health of the animals remained good. One month later these rabbits, and also control rabbits, received intravenous injections of 2 c.c. of a virulent culture of the same bacillus pyocyaneus. The controls died in a few hours with the typical symptoms and lesions. The vaccinated animals developed symptoms of a less intense character—fever, diuresis, diarrhœa—but after a few hours these symptoms subsided, the animals took food, and completely recovered.

### Chronic Nephritis and the Suprarenals.

There is a good deal of uncertainty and dispute in regard to conditions of chronic nephritis and the pathological relationship of the hyperplasia of the suprarenal capsules often found present in such cases. A certain number of authorities have endeavoured to show that in chronic nephritis, with or without atheroma, an adenomatous hyperplasia of the suprarenals is a constant phenomenon, whereas it is only exceptionally present in cases of nephritis without hypertension, and they have concluded from this a causal relation between this suprarenal hyperplasia and the hypertension. On the other hand, it has been contended that these anatomo-pathological lesions involve only the cortical part of the suprarenals, the medullary portion being hardly affected, so that there is no evidence of an increase in the production of adrenalin, the supposed cause of vascular degeneration and hypertension. Dr. d'Alessandro, of Sienna, has recently studied the subject and bases his conclusions on observations of eleven cases of chronic nephritis with five autopsies. He considers that the supposed causal relation in cases of chronic nephritis between the hyperfunction of the suprarenals, on the one hand, and the hypertension, cardiac hypertrophy, arterio-sclerosis, and atheroma on the other, is highly problematic and far from proved. He points out that the suprarenal changes found in cases of chronic nephritis are analogous to those found in the infectious diseases and in those of advanced age. They are entirely different from those changes presented by the capsules in a condition of hyperfunction. They are, therefore, a secondary phenomenon and dependent upon the primary pathological changes in the whole organism, set up by different causes in different cases (infections, toxi-infections, etc.).

### Nephro-ureterectomy.

In the *Annals of Surgery* Dr. Lilienthal pleads for a revision of the ordinary technique of nephrectomy by the addition of ureterectomy, a detail which he considers to be of great importance. In a number of cases he has removed the ureter down to the bladder itself by a method so simple and attractive that he thinks it will at once recommend itself to surgeons. The extra time required is said to be only five or ten minutes. After performing extra-peritoneal nephrectomy by any of the approved methods, with separate treatment of the ureter and the vessels, the former is cut between two ligatures or forceps, and the mucous membrane of the stump cauterised with 95 per cent. phenol. The ureter is now drawn out of the wound if possible, or it is isolated with gauze; the forceps or ligature is removed, and a good-sized flexible *urethral* bougie with a conical or olive point is passed down towards the bladder. A ligature is tied tightly round the ureter and the instrument, so as to hold the bougie in place and prevent the leakage of infected fluids from the canal. The greater part of the lumbar wound is then closed with drainage in the usual manner, and the patient turned on to his back. An oblique incision, 1½ to 3 inches in length, according to the thickness of the abdominal wall, is made about an inch to the median side of the anterior superior iliac spine. This is carried down to the peritoneum, and the gloved finger is pushed down extraperitoneally to the ureter, which is then easily separated and drawn with the bougie out of the wound. The bougie can then be withdrawn and the ligature pulled tight. The ureter is next followed right down to the bladder, tied and cut, and the stump disinfected with phenol. The wound can then be closed with drainage for forty-eight hours. This operation is devised to avoid the danger of leaving a suppurating or tuberculous ureter behind after nephrectomy, and thus to make that operation both more surgical and more successful.

### To Remove Adhesive Plaster.

A useful hint is given by Dr. Beardsley in the *Journal of the American Medical Association* on the removal of adhesive plaster. Everyone knows how painful this is to a patient who has had a strip of surgeon's plaster applied to a part which is hairy. Even when a preliminary shaving has been done the hairs have often grown again when the time comes for the plaster to be removed, and the process is an unpleasant one for both doctor and patient. Dr. Beardsley thinks very little of the usual methods of facilitating removal; turpentine is messy and only partly effective, benzine, alcohol, ether, and peroxide of hydrogen are also inefficient. He puts great reliance, however, in oil of wintergreen, which dissolves very rapidly and successfully the resinous adhesive ingredients of the ordinary plaster. It is advisable to use a small quantity only of the oil, for it quickly soaks through the fabric and diffuses itself along the material. He also especially advocates, when very large areas of plaster are being dealt with, an ointment containing 10 per cent. of oil of wintergreen in adeps lanæ hydrosus; this is even more successful than the pure oil.

### Obesity and Uterine Carcinoma.

Practising obstetricians frequently remark that hysterectomy for malignant disease of the cervix is frequently rendered difficult by the adipose deposits of the patient; much more often comparatively than in hysterectomy for fibroids, for instance. Whether there is really any connection between obesity and malignant disease of the cervix is a very doubtful point; conceivably the explanation might lie in the common factor of repeated gestations, which dispose to both conditions. Dr. Atlee, of Philadelphia, has no doubts whatever as to the fact of this association; indeed, he propounds a new help in diagnosis of early cases founded on it. Excluding, of course, cases in which malignant cachexia has supervened in long-standing cases, the author attaches great importance to the date when the patient's maximum weight was attained. If this happened before, and lasted up to, the beginning of the onset of suspicious symptoms in a middle-aged woman, he regards it as a strong point in favour of malignancy. The existence of maximum weight is not alone, he grants, sufficient to determine the presence of uterine cancer; associated with maximum weight in the incipient stages of this malady will be found also unexcelled health and strength, often rapidly attained subsequent to previous conditions of imperfect health. Concurrent with these features of suddenly acquired health will appear mental and physical apathy, together with a rather abnormal inclination to sleep. Cases are quoted in support of these contentions, which apply only to malignant disease of the uterus, not to that of other parts. Without calling in question the intentions of the author, it must still be admitted that he is a long way from proving his case.

### Statistics of Anti-enteric Inoculation.

Colonel R. H. Firth, of the Royal Army Medical Corps, is a firm believer in the value of anti-typhoid inoculation as now perfected, and in the *Journal* of his regiment he analyses the typhoid statistics of the British Army in India in support of his views. It is quite clearly proved that both the incidence and the mortality from this disease among our troops have been enormously reduced in recent years. The author takes fully into account the many causes which have contributed to this improvement, such as segregation of all drafts and new arrivals, establishment of convalescent hospitals, detection and treatment of "carrier" cases, and the greater attention of all ranks to sanitation and hygiene. But after allowing for all these factors he believes that the case for inoculation is a strong one, whether from the point of view of freedom from attack or of recovery after attack. He advocates the adoption of steady and persistent reinoculation of all men who have been inoculated more than thirty months, a system which, as a footnote informs us, has just been officially put into operation by the principal medical officer in India. In 1910, it is satisfactory to note, the admission rate of European troops in India for typhoid fever was 4.6 per 1,000; and the death-rate only 0.63. If these figures can be still further reduced it will be a great achievement. Unfortunately we understand that 1911 has not begun well so far, and is likely to show figures not quite so good as these.

### Otosclerosis and Pregnancy.

The influence of pregnancy upon chronic ear disease is a subject to which neither obstetricians not otologists have paid sufficient attention, in the opinion of Dr. Brickner, who writes to the *American Journal of Obstetrics and Gynæcology* upon the connection of the two. In the main his remarks apply to chronic progressive deafness of the variety known as otosclerosis; but he believes that pregnancy has deleterious effects upon hearing in certain other diseases also. Admitting that the pathological processes involved are not at all clear, he still pronounces that pregnancy has a very bad effect upon otosclerosis; for the deafness increases immediately upon the advent of pregnancy, grows worse during gestation, and remains permanently worse after delivery than it was before pregnancy began. Repeated pregnancies have the effect of rendering the hearing progressively worse. The interruption of pregnancy may preserve what hearing then exists; but it may accomplish no more than this, and the hearing may subsequently continue to deteriorate. Dr. Brickner further suggests that it is right to induce abortion early in pregnancy after it has been established unquestionably that the pregnancy is causing the hearing to become diminished. The right to render these women sterile, he adds, depends on individual cases and is a debatable question.

### Meat Inspection.

At the annual Congress of the Royal Sanitary Institute, held in Belfast last week, somewhat severe strictures were passed on the system of meat inspection in this country. It was pointed out by several speakers that only about half the total consumption of meat was supplied by home-killed cattle; last year over seven million hundredweight of frozen and chilled beef was imported. Mr. J. A. Jordan, of Belfast, deplored the fact that in many small towns there was practically no inspection, and unscrupulous butchers make a practice of going into these rural districts and slaughtering their cattle in those places where they were left to do as they liked. As the only practical means of insuring the efficient inspection of meat shops it was advocated that all meat exposed for sale should be marked distinctly. At the present time the inspectors had no means of knowing whether the meat exposed for sale in shops had been previously inspected or not. In fact, Mr. W. G. Barnes, of the Metropolitan Cattle Market, asserted that more than half of the meat consumed in this country was never seen by an inspector. In the course of a discussion Dr. Collingridge, the Medical Officer of Health of the City of London, pointed out that it was not so necessary that foreign meat should be branded as it was that sound, healthy meat produced at home should be distinctly marked. It was unavoidable that the consumer must, to a great extent, trust the butcher; the essential thing was for a proper watch to be kept by the local authority. A reassuring statement was made by Dr. W. Hanna, of the Port of Liverpool, on the condition of foreign meat, particularly in regard to pork. He was fully satisfied, after searching examinations, that the pigs imported from China were as sound as any bred in this country, and as the result of a stricter system of inspection in that country considerable improvement as regards disease had occurred in these imported pigs.

